# Synthesis and Characterization of Sulfur and Sulfur-Selenium Nanoparticles Loaded on Reduced Graphene Oxide and Their Antibacterial Activity against Gram-Positive Pathogens

**DOI:** 10.3390/nano12020191

**Published:** 2022-01-07

**Authors:** Rashmi Niranjan, Saad Zafar, Bimlesh Lochab, Richa Priyadarshini

**Affiliations:** 1 Department of Life Sciences, School of Natural Sciences, Shiv Nadar University, Gautam Buddha Nagar 201314, India; rn161@snu.edu.in; 2 Materials Chemistry Laboratory, Department of Chemistry, School of Natural Sciences, Shiv Nadar University, Gautam Buddha Nagar 201314, India; sz539@snu.edu.in

**Keywords:** sulfur-selenium/reduced graphene oxide nanomaterials, antibacterial, oxidative stress

## Abstract

Resistance to antimicrobial agents in Gram-positive bacteria has become a major concern in the last decade. Recently, nanoparticles (NP) have emerged as a potential solution to antibiotic resistance. We synthesized three reduced graphene oxide (rGO) nanoparticles, namely rGO, rGO-S, and rGO-S/Se, and characterized them using X-ray diffraction (PXRD), Raman analysis, and thermogravimetric analysis. Transmission electron microscopy confirmed spherical shape nanometer size S and S/Se NPs on the rGO surface. Antibacterial properties of all three nanomaterials were probed against Gram-positive pathogens *Staphylococcus aureus* and *Enterococcus faecalis*, using turbidometeric and CFU assays. Among the synthesized nanomaterials, rGO-S/Se exhibited relatively strong antibacterial activity against both Gram-positive microorganism tested in a concentration dependent manner (growth inhibition >90% at 200 μg/mL). Atomic force microscopy of rGO-S/Se treated cells displayed morphological aberrations. Our studies also revealed that rGO composite NPs are able to deposit on the bacterial cell surface, resulting in membrane perturbation and oxidative stress. Taken together, our results suggest a possible three-pronged approach of bacterial cytotoxicity by these graphene-based materials.

## 1. Introduction

Bacteria play an important role in human health and environment. Antimicrobial agents including antibiotics have been used to treat bacterial infections due to their ease of access, cost effectiveness, and faster results. However, overuse and misuse of antibiotics as therapeutic agents has led to development/evolution of antibiotic resistance in bacterial species [1,2]. Primarily, selection pressure results in propagation of mutations, which are beneficial for the bacterial growth [3]. Adaptive benefits from small mutation supplies an antibiotic resistance enzyme [4]. Indiscriminate use of antimicrobial agents in agricultural, veterinary, and human clinical setting provides an optimum setting for selection of resistant bacteria. Once a beneficial mutation is “selected”, the same can be transferred to other species in nature [5]. Lateral transfer and accumulation of resistance phenotypes lead to increased bacterial resistance to antibiotics and development of “super-bacteria” or multidrug-resistant bacteria (MDR). Development of such drug-resistant bacteria in clinical settings leads to nosocomial infections, which are a significant problem to global health [6]. In 2013, the CDC (the Centers for Disease Control and Prevention) declared that we have entered the “post- antibiotic era” [7,8]. Fifteen MDR bacteria have been declared a substantial threat to U.S. public health and national security by the IDSA, the Institute of Medicine, and the federal Interagency Task Force on Antimicrobial Resistance [9]. Prime examples of MDR among Gram-positive pathogens are methicillin-resistant *Staphylococcus aureus* (MRSA) and vancomycin-resistant *Enterococcus* species (VRE) [10]. Though *S. aureus* is mainly present on skin and mucus membranes, it can spread throughout the body in the bloodstream, causing serious infections under clinical settings [11,12]. The mortality rate because of MRSA in America is more than the combined mortality rate due to AIDS, Parkinson’s disease, and emphysema [9]. Similarly, VRE infections include bloodstream and urinary tract infections [13]. Infection with VRE has been to shown to result in longer hospital stay and are also associated with enhanced mortality [14]. Apart from these, MDR includes drug-resistant *Mycobacterium tuberculosis*, carbapenem-resistant *Enterobacteriaceae* (CRE), MDR *Pseudomonas aeruginosa*, MDR *Acinetobacter*, and (extended-spectrum beta-lactamases) ESBL-Producing *Enterobacteriaceae* [15]. This evolutionary development of “superbugs” has pushed us to search for new alternative therapeutic approaches to tackle problems caused by bacteria [16]. With the advent of technology and research, nanomaterials have emerged as an alternative to antibiotics. Antibiotics majorly target cell-wall synthesis, translation, and DNA replication in bacterial cells. Overuse of antibiotics has led to bacterial resistance against these modes of action by expression of antibiotic degrading enzymes, modification of cell components, and change in membrane permeability [17]. Therefore, NPs have an advantage as antimicrobial agents since their mode of action involves physical contact with the cell followed by oxidative stress generation, metal ion release, or non-oxidative mechanisms. The large surface-area-to-volume ratio of NPs provides increased contact area with the organisms, enhancing its potential as an alternative to traditional antibiotics [17,18]. Antimicrobial activity of NPs is influenced by physicochemical properties of NPs such as their size, charge, zeta potential, surface morphology, crystal structure, composition, and method of preparation. Other factors including environmental conditions such as aeration, pH, temperature, strain, physiological state of the bacteria, and the exposure time also play a part in the activity of nanomaterials. Even the mechanism of action might vary with varying microbes because of the differences present in genetics, cell-wall structure, and metabolic pathways among bacteria [1]. As of now, nanomaterials in use as antimicrobial agents involve metal, metal oxides, and organic NPs. Among these, metal oxides NPs exhibit maximum activity against bacteria [19,20].

Since the ancient era, organic- and inorganic-sulfur and selenium compounds have been widely used to prevent and treat a variety of infections caused by microorganisms. Among chalcogens, both S and Se have reported medicinal uses [21] and the potential to control several biological applications [22,23]. The key constituents in these compounds are sulfur and selenium, which are considered to affect several biological activities, including antimicrobial, antioxidant, and anticancer, etc. [24]. However, multi-step synthesis of such materials, and raising concerns about tackling antibiotic resistance, environmental pollution, relatively complex processing, and high cost hampers industrial viability. On the contrary, elemental sulfur is produced in large quantities as an industrial waste from petroleum industry. However, only bare sulfur cannot be used as such due to its meagre water solubility and bulk nature, thus limiting biological applications.

Several studies have demonstrated fixation of sulfur and selenium into inorganic forms or as such with conversion and miniaturization in the form of nanoscale improves antibacterial activity [25,26,27,28]. Sulfur NPs demonstrated antibacterial activity of sulfur NPs against both Gram-positive (*Staphylococcus aureus*) and multidrug-resistant Gram-negative (*Escherichia coli* and *Pseudomonas aeruginosa*, *Klebsiella pneumoniae*, *Acinetobacter baumannii*, *Stenotrophomonas maltophilia*, and *Enterobacter aerogenes*) bacteria [29,30,31,32]. These NPs impaired the functioning of mitochondrial enzymes involved in cellular respiration and oxidative phosphorylation, and led to a decline in bacterial growth. The inhibitory effect of selenium NPs on *S. aureus* at an early time (up to 5 h of contact) may prevent biofilm formation [31]. Shape, size, and morphology of NPs, and the nature of the capping agent loading on substrate are known to influence antibacterial performance [32,33,34,35].

Among synthetic carbon nanomaterials, graphene oxide (GO) and reduced graphene oxide (rGO) exhibit strong antibacterial activity [36]. They induce membrane stress and physical damages on cell membranes due to sharp edges of graphene nanosheets, leading to the loss of bacterial membrane integrity and the leakage of RNA. Additionally, wrapping of graphene nanosheets over bacteria and existence of surface functionalities enhances oxidative stress and prevents its proliferation [37,38]. The conductive nature of rGO and extent of surface roughness hamper the bacterial growth [39,40].

There is a significant growing interest in individual study on S, Se, and graphene nanomaterials for antibacterial applications. A combination of these NPs can profoundly affect the growth of bacteria and may strongly influence antibacterial activities. In the current work, we synthesized rGO, rGO-S, and rGO-S/Se NPs by convenient and cost-effective methods. The nanomaterials were characterized and studied and their performance was compared against two Gram-positive bacteria pathogens (*S. aureus* and *E. faecalis*).

The time and concentration-dependent antibacterial activities of NPs were determined using Minimum Inhibitory Concentration (MIC) and colony-forming unit (CFU) assay. The mechanism of bacterial toxicity was determined by various methods such as AFM analysis, Ellman assay, and in vitro ROS measurements. Our studies revealed that rGO composite NPs deposit on the bacterial cell surface and cause membrane perturbation and oxidative stress, suggesting a three-step bacterial-cytotoxicity mechanism. We further evaluated the antibacterial activity of NPs against Gram-positive pathogens *S. aureus* and *E. faecalis* and observed the antagonistic cellular interactions of S/Se NP’s with Gram-positive pathogens. Understanding the nature, size, and hybridization of NPs profoundly affected the antibacterial performance and the present study may provide guidelines to design new materials especially to combat MDR bacteria and assist in designing novel coating materials.

## 2. Materials and Methods

*Materials*: Natural graphite flakes (Alfa Aesar, Haverhill, MA, USA, 99.8%, 325 mesh), sulfur powder (Alfa Aesar, Haverhill, MA, USA, 99.5%, sublimed, 100 mesh), selenium powder (Sigma Aldrich, St. Louis, MO, USA, 99.5%, 100 mesh), ethylenediamine (EDA, Alfa Aesar, Haverhill, MA, USA, 99%), fluorescein isothiocyanate (FITC, TCI Chemicals India Pvt. Ltd., Chennai, India), ethanol (99.9%), sulfuric acid (Finar, Ahmedabad, India), sodium nitrate, potassium permanganate, and hydrogen peroxide (30% *w*/*w*, aqueous) were obtained from Fisher scientific (Mumbai, India). All reagents were used as received.

*Characterization of Nanomaterials*: Transmission electron microscopy (TEM, Akishima, TYO, Japan) images were recorded using an HRTEM, JEOL-2100F instrument under an accelerating voltage of 200 kV, and samples for TEM analysis were prepared by dispersing 1 mg of the material in 5 mL of ethanol solution followed by ultrasonication for 5 min and dropwise addition onto a copper grid. Elemental mapping was recorded using a Scanning Electron Microscope (SEM) (JEOL, Akishima, TYO, JPN) equipped with energy dispersive X-ray (EDX) (AMETEK, Brewyn, PA, USA). Powder X-ray diffraction (PXRD, Billerica, MA, USA) was recorded using a Bruker D8 diffractometer, using Cu Kα (λ = 1.5406 Å) radiation. Thermogravimetric analysis (TGA, Columbus, OH, USA) was recorded using a Mettler Toledo instrument under N_2_ atmosphere (40 mL min^−1^) at a heating rate of 10 °C min^−1^. Atomic force microscopy (AFM) (PARK, XE-007, Gwanggyo-ro, Suwon, KR) in noncontact mode was used to detect the morphology of bacteria. Raman spectroscopy (Kotoku, TYO, Japan) measurements were carried out with an STR500 Airix microscope using a 532 nm laser at a power of 3 mW.

### 2.1. Synthesis of Graphene Oxide (GO)

GO was prepared by a modified Hummer’s method [41]. In brief, to a stirring mixture of concentrated sulfuric acid (70.0 mL, 1.26 mol) and sodium nitrate (1.5 g, 17.65 mol) at 0 °C, graphite flakes (3.0 g) were added slowly while maintaining the temperature at 0 °C. Once the addition was completed, potassium permanganate (9.0 g, 45.65 mol) was gradually added by close monitoring and maintaining the temperature below 20 °C. After the addition, the ice bath was removed, and the reaction mixture was stirred for 30 min. The color of the solution turned from black to a grey-brown color in 30 min., after which distilled water (140.0 mL) was added. The reaction was then heated at 98 °C for 15 min. and the color changed to dark brown color. The reaction was allowed to cool to room temperature before the addition of water (420.0 mL) and hydrogen peroxide (30%, 3 mL, 0.03 mol) to produce a brown-colored suspension. The so-formed GO was filtered, and the residue was washed exhaustively with water (7 × 500 mL) to remove the acidic impurities. The residue was sonicated and stored in water for 6 h until the pH of the filtrate was reconfirmed as neutral. The residue of GO was dried under vacuum to produce GO as a brown solid (1.74 g, 58%).

### 2.2. Synthesis of Reduced Graphene Oxide (rGO)

GO (500 mg) was dispersed in deionized water (300 mL) by bath sonication for 20 min. Once dispersed, aqueous ammonia (25% in water, 120 μL, 1.74 mmol) was added to obtain the pH value of 12 (checked with pH meter, Horiba, Kyoto, Japan). Hydrazine hydrate (166 μL, 3.42 mmol) was then added to the mixture followed by stirring at 95 °C for 12 h in a nitrogen atmosphere. After cooling to room temperature, the so obtained rGO was filtered and the residue was subjected to repetitive washing with deionized water (2 × 250 mL) followed by acetone (15 mL). The obtained product was dried under vacuum to give rGO as a black solid (150 mg, 30%).

### 2.3. Synthesis of rGO-S Composites

In a vial containing sulfur (S, 3.0 g, 93.56 mmol), EDA (40 mL, 592 mol) was added under a nitrogen atmosphere and tightly closed. The contents were kept at vortex for 5 min to obtain a dark red S-EDA precursor solution. In another flask, rGO (300 mg) was dispersed in a solvent mixture of distilled water and ethanol (10:1, 660 mL) under bath sonication (Branson Sonics, Brookfield, CT, USA) for 60 min, and the pH of the mixture was determined as 6.6. To a vigorously stirred rGO dispersion, S-EDA precursor solution was added dropwise at a rate of ~1 mL min^−^^1^. After the completion of the addition, the reaction mixture solution was further stirred for 30 min under airtight conditions. Then the mixture was filtered, and the residue was rinsed with deionized water (5 × 100 mL) first until no red-yellow color filtrate was obtained. Thereafter residue was washed with alcohol (3 × 15 mL) several times. Finally, the residue was dried at 60 °C for 12 h in a vacuum oven to obtain rGO-S composite (1.6 g, 49%) as a black powder [42].

### 2.4. Synthesis of rGO-S/Se Composites

A physical homogeneous blend of sulfur (2.1 g, 65.6 mmol) and selenium (0.9 g, 11.4 mmol) powder (S:Se, 7:3 *w*/*w*, 3.0 g) was prepared by grinding the two together in a mortar and pestle for 15 min. The resultant black color powder was then treated using the same methodology adopted for the preparation of rGO-S composite. In this composite preparation, a green color filtrate was obtained. Finally, the rGO-S/Se composite was obtained as a green-black color solid (1.95 g, 59%) after drying at 60 °C in a vacuum oven for 12 h.

### 2.5. Bacterial Strains, Medium, and Growth

Gram-positive bacteria *Staphylococcus aureus* UAMS-1 (kindly provided by Dr. Vance G. Fowler, Duke University, USA) and *Enterococcus faecalis* (*E. faecalis*) lab strain [43] were used in this study. The bacterial strains were propagated on tryptic soy broth (TSB) (HiMedia, Mumbai, India) agar plates at 37 °C under aerobic conditions.

### 2.6. Antibacterial Activity of NPs

For in vitro antibacterial activity of NPs, 1:100 dilution of overnight-grown *S. aureus* and *E. faecalis* was inoculated in 24-well plates in TSB containing varying concentrations of nanomaterials. As the NPs synthesized did not show any statistically significant inhibition below 100 μg/mL concentrations, rGO and rGO-S at the concentrations of 100 μg/mL, 150 μg/mL, and 200 μg/mL and rGO-S/Se at 100 μg/mL, 150 μg/mL, 180 μg/mL, and 200 μg/mL were incubated in an orbital shaker at 37 °C for 9 h with shaking at 170 rpm. The wells containing only *S. aureus* and *E. faecalis* without NPs were used as negative controls. S, Se, and S/Se NPs at three different concentrations of 100 μg/mL, 150 μg/mL, and 200 μg/mL were also tested for their antibacterial activity against *S. aureus*. All NPs were pre-sterilized before assays using UV treatment for 30 min. After incubation, the antibacterial effect of NPs was assayed by measuring optical density (OD) using i-Mark Microplate reader (Bio-Rad, USA) at 600 nm wavelength (OD_600_). OD_600_ was used for estimating the concentration of bacteria or other cells in a liquid sample. Since the 600 nm wavelength does not hinder the bacterial growth, it is the most commonly used spectrophotometric technique. Bacterial growth inhibition percentage was calculated by the Equation (1):

(1) $$\;\%\;\mathrm{inhibition}=\frac{OD\left(a\right)-OD\left(b\right)}{OD\left(a\right)}\times 100$$

where (*a*) is OD of *S. aureus* at 600 nm and (*b*) is OD of *S. aureus* incubated with nanomaterials determined at 600 nm.

*S. aureus* treated with rGO-S/Se at the concentration of 200 μg/mL was 10-fold serially diluted. Finally, 100 μL bacterial solution of 10^−4^, 10^−5^, and 10^−7^ dilution were spread on TSB plates and incubated overnight for colony forming unit (CFU) assay. For the study of the bacteriostatic effect of NPs, 1:100 diluted overnight grown cultures of *S. aureus* were diluted 1:100 and incubated with 70, 100, 150, and 200 μg mL^−1^ concentrations of rGO-S/Se under aerobic conditions at 37 °C. OD of the bacterial suspension was measured using a spectrophotometer (Eppendorf) at 0, 2, 4, 6, 8, 11, 18, and 21 h after treatment with NPs [44].

### 2.7. Ellman’s Assay

The concentration of thiols in reduced glutathione (GSH) was quantified by the Ellman’s assay using Ellman’s reagent (5,5′-dithiobis (2-nitrobenzoic acid) DTNB, Alfa aesar) with nanoparticles only with certain modifications [43]. Ellman’s assay is a rapid, sensitive, and reproducible method for the determination of free thiol groups of GSH. Nanomaterials rGO (70 μg/mL, 100 μg/mL, 150 μg/mL, and 200 μg/mL), rGO-S (100 μg/mL, 120 μg/mL, 150 μg/mL, 180 μg/mL, and 200 μg/mL), and rGO-S/Se (70 μg/mL, 100 μg/mL, 120 μg/mL, 150 μg/mL, 180 μg/mL, and 200 μg/mL) dispersions in water were taken at varying concentrations in 50 mM bicarbonate buffer (pH 8.6) and incubated with 0.8 mM GSH for 3 h at 150 rpm in the dark. After incubation, 0.05 M Tris-HCl and 100 mM DTNB (Ellman’s reagent) were added to the mixture leading to the formation of yellow 2-nitro-5- thiobenzoic acid (TNB) product. The absorbance of samples was recorded at 412 nm with a microplate reader (Tecan). A GSH solution without NPs was used as the negative control, whereas GSH oxidation by hydrogen peroxide (H_2_O_2_) (10 mM) was used as the positive control in the experiments [39,44,45]. All the experiments were performed in triplicates. The loss of GSH was calculated using Equation (2):

(2) $$\mathrm{Loss}\;\mathrm{of}\;\mathrm{GSH}\;\%=\left[\frac{\left(absorbance\;of\;negative\;control-absorbance\;of\;sample\right)}{absorbance\;ofnegative\;control}\;\right]\times 100$$

### 2.8. ROS Assay

Intracellular ROS level was detected using active oxygen detection probe 2′,7′-dichlorodihydrofluorescein diacetate (DCFH-DA) (Thermo Fischer Scientific, Waltham, MA, USA) in bacterial cells. Dichlorodihydrofluorescein-diacetate is a fluorogenic dye that, on diffusion in to the cell, is cleaved by intracellular esterases, converting it to a non-fluorescent compound which, on reaction wih ROS, is rapidly oxidized to highly fluorescent 2′,7′-Dichlorodihydrofluorescein. In the absence of ROS no fluorescent signal is observed. Overnight grown culture of *S. aureus* and *E. faecalis* was diluted 1:100 in fresh TSB medium and incubated further to obtain an OD_600_ in the range of 0.4–0.5. The bacterial culture was then centrifuged at 7000 rpm for 5 min followed by washing with phosphate-buffered saline (PBS) (pH 7.4) (Sigma-Aldrich, St. Louis, MO, USA) thrice [46]. Finally, the pellet was resuspended in PBS and treated with 200 μg/mL of rGO-S/Se nanomaterials for 8 h (2000 rpm, 37 °C). Bacterial suspensions without NPs exposure were taken as negative controls. After treatment, the bacterial suspension was incubated with DCFH-DA for 30 min following the manufacturer’s protocol. Excess staining was removed by washing with PBS and cells were observed by fluorescence microscopy using A1 confocal Galvano microscope (Nikon, Japan) equipped with a Nikon camera using Plan Apo 100X/1.40 oil objective and fluorescence intensity was measured using ImageJ software. For measurement of DCF intensity, we selected oval ROIs (region of interest) around the bacterial shape and used the mean intensity tool in ImageJ [46,47,48].

### 2.9. Atomic Force Microscopy

Morphological changes in bacteria caused by nanomaterials exposure were detected by atomic force microscopy (AFM). For AFM analysis, bacterial cells in the exponential phase were harvested and resuspended in PBS and exposed to 200 μg/mL of rGO-S/Se nanomaterials for 8 h (200 rpm, 37 °C). Bacterial suspension without treatment was taken as the negative control. After exposure with NP, the suspensions were centrifuged for 10 min at 5000 rpm and 10–15 μL of bacterial suspension was deposited on cover glass and air-dried [27,49,50]. All the samples were prepared in triplicates under the same conditions and were observed with AFM (PARK, XE-007) in noncontact mode.

### 2.10. FITC (Fluorescein Isothiocyanate) Labelled NPs Detection

An aqueous solution of FITC (50 μg/mL, 5 mL) was added to rGO/S-Se (5 mg) [51]. The mixture was kept for 3 h in bath sonication. The mixture was kept for 3 h in bath sonication to form FITC-labelled rGO/S-Se nanocomposite. The prepared sample was used for further analysis. Interaction studies of rGO-S/Se-FITC in bacterial cells were carried out using confocal microscopy. For this, the overnight culture of S. aureus was diluted at 1:100 and grown to an OD_600_ value of 0.5. The bacterial cells were harvested by centrifugation at 7000 rpm for 5 min at room temperature. The pellets obtained were resuspended in PBS and incubated with rGO-S/Se-FITC (100 μg/mL) for 8 h. After incubation, the bacterial cells were pelleted down and washed and then observed by fluorescence microscopy using an A1 confocal Galvano microscope (Nikon, Japan) equipped with a Nikon camera using Plan Apo 100X/1.40 oil objective [29].

### 2.11. Statistical Analysis

All experiments were performed in triplicate, and the data were expressed as the mean and standard deviation. A two-tailed *t*-test was used for statistical analysis and significant values were claimed when the *p*-value value was lower than 0.05.

## 3. Results and Discussion

### 3.1. Synthesis of Nanocomposites

**Figure 1:**
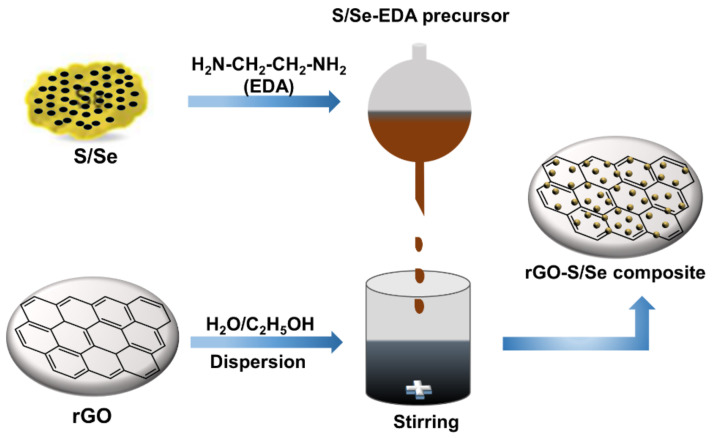
Schematic illustration of the synthetic procedure. Deposition of sulfur/selenium nanoparticles (S/Se NPs) on rGO via the sulfur-amine chemistry.

S NPs on reduced graphene oxide (rGO) sheets were prepared via the sulfur-amine chemistry as illustrated in the chemical reaction (Equation (3)) [52]. As shown in the equation, nucleophilic attack of amine group in EDA opened the S_8_ ring of to form linear chain alkylammonium polysulfides [53,54] with an alkyl ammonium counter ion as shown in Equation (3a). The sulfur-ethylenediamine complex precursor was added dropwise to the rGO dispersion in water/ethanol mixed solvent as illustrated in Figure 1. This led to the decomposition of the sulfur-amine complex to release elemental sulfur particles, which nucleate on the surface of rGO sheets as shown in Equation (3b).

(3a) $$2\left({R\text{-}\mathrm{NH}}_{2}\right)+\;S_{8}\;\to \;\left(R\text{-}{{\mathrm{NH}}_{3}}^{+}\right)\left(\mathrm{RNH}\text{-}{S_{8}}^{-}\right)$$

(3b) $$\left(R\text{-}{{\mathrm{NH}}_{3}}^{+}\right)\left(\mathrm{RNH}\text{-}{S_{8}}^{-}\right)\;\overset{H^{+}}{\to }\;\left(R\text{-}{{\mathrm{NH}}_{3}}^{+}\right)+\;S_{8}$$

It was reported the formation of monodisperse S NPs is governed by the reaction conditions such as time, rate of addition of S-EDA precursor, and pH of the solution [42]. Likewise, the rGO-S/Se composite was also prepared.

### 3.2. Characterization of Nanocomposite

**Figure 2:**
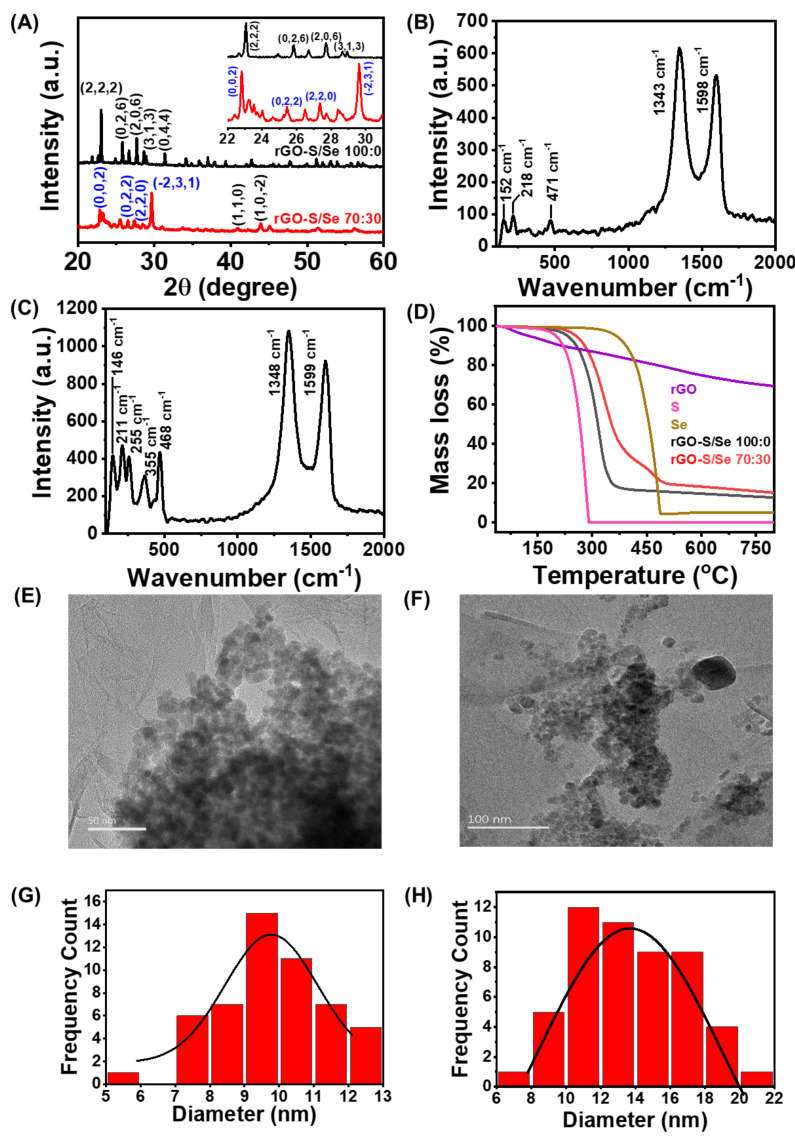
Characterization of NPs: Powder X-ray diffraction (**A**) analyses of rGO-S and rGO-S/Se composite (inset shows the zoomed-in region); Raman spectra of rGO-S (**B**) and rGO-S/Se (**C**); TGA curves (**D**); Representative TEM images of S ((**E**), 50 nm scale) and S/Se NPs ((**F**), 100 nm scale) present on rGO and (**G**,**H**) particle size distribution of S and S/Se NPs (non-aggregated state, *n* = 55) on rGO.

To confirm the deposition of S and S/Se on rGO, powder X-ray diffraction (PXRD) of the prepared nanocomposites rGO-S and rGO-S/Se was recorded. Figure 2A shows that the sulfur on the rGO-S composite exhibited the same XRD pattern as pure elemental S (Figure S1), indicating the methodology adopted to load sulfur onto the rGO did not induce any changes in the crystal structure of bulk S [42]. The XRD plot of rGO-S/Se indicated the formation of S-Se nanoparticle alloy with a monoclinic structure as marked with the indices in blue color. The additional 2θ peaks with the indices in black color are attributed to the mainly pure selenium NPs (hexagonal Se symmetry) [55]. However, a small shift in these 2θ values to a lower value was noticed, which can be accounted for the variation in chalcogenide chains due to hybridization of S within segments rich in Se-Se arrangements [54].

Raman analysis was performed to support the existence of chalcogenide NPs and the hybridization of S and Se using solution-based chemistry. Figure 2B,C shows Raman spectra of both rGO-S and rGO-S/Se, which revealed sharp and strong Raman peaks due to rGO at 1343 and 1598 cm^−1^ due to the D and G bands respectively. The existence of S NPs was confirmed by the appearance of peaks at 152, 218, and ~471 cm^−1^ due to S-S phonon vibrations. The rGO-S/Se composite showed phonon modes at 146 cm^−1^ attributed to Se-Se bond vibrations. Additionally, the peaks at 211, 255, and 355 cm^−1^ were assigned to S-Se bonds along with 468 cm^−1^ due to S-S bonds. The intensity ratio of D and G bands i.e., *I*_D_/*I*_G_ was calculated as 1.17 and 1.21 for rGO-S and rGO-S/Se, respectively, suggesting rGO present in nanocomposite has a more graphene-like structure and defects are induced due to the presence of S and S-Se NPs on rGO surface [54].

To determine the amount of chalcogen NPs loading onto the rGO, thermogravimetric analysis (TGA) was performed as shown in Figure 2D. TGA plot of rGO showed a steep mass loss of ~25% when heated from room temperature to 800 °C. Pristine S and Se material showed a one-step decomposition with a significant mass loss at 281 °C and 484 °C, respectively, signifying higher volatility of sulfur than selenium. Thermal stability of the S/Se-based rGO composite lied in between pure S and Se. The rGO-S and rGO-S/Se composites degraded at 320 °C and 335 °C, respectively. A higher temperature decomposition of deposited S NPs than bare S is suggestive of significant interaction between the rGO and loaded NPs. The incorporation of Se in the hybrid S/Se NPs enhanced the thermal stability as compared with S particles loaded on rGO due to the higher thermal stability of selenium. The TG analysis confirmed a high loading of sulfur and sulfur-selenium content in the rGO-S and rGO-S/Se composites, determined as 84% and 82% respectively.

Transmission electron microscopy (TEM) images as shown in Figure 2E,F confirmed the spherical shape nanometer size S and S/Se NPs on the rGO surface. Interestingly, on the rGO surface, S/Se NPs revealed less aggregation compared with S NPs. The average size of the particles was determined as 9.5 and 12.0 nm for S and S/Se NPs, respectively. The particle size distribution analysis histograms, Figure 2G,H, were calculated using ImageJ software using the number of particles in a non-aggregated state (*n* = 55). The existence of a narrow particle size distribution of synthesized NPs confirmed successful loading of sulfur and sulfur-selenium based NPs on rGO.

### 3.3. Antibacterial Activity of rGO-S/Se NPs

**Figure 3:**
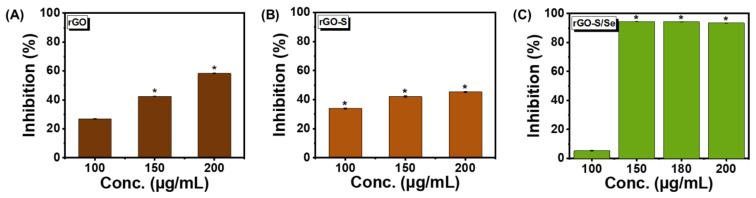
Effect of rGO, rGO-S, and rGO-S/Se on *S. aureus* growth. Percentage of bacterial growth inhibition when exposed to varying concentrations of (**A**) rGO; * *p*-value < 0.05, (**B**) rGO-S; * *p*-value ≤ 0.01 and (**C**) rGO-S/Se; * *p*-value < 0.0001. Error bar represents the standard error of the mean.

We chose *Staphylococcus aureus* gram-positive cocci as the primary model organism to test the antibacterial activity of NPs. *S. aureus* is known to cause skin, respiratory, bone, and endovascular infections, and also life-threatening bacteraemia, sepsis, and toxic shock syndrome [11,12]. As evident from Figure 3, all NPs revealed a concentration-dependent antibacterial activity when analyzed at the same concentration range. rGO-S/Se was found to be most effective with more than 90% inhibition observed at and above concentrations of 150 μg/mL (Figure 3C). In comparison, rGO and rGO-S NPs only showed 43% and 42% at 150 μg/mL concentration (Figure 3A,B). This suggested that rGO-S/Se have more antibacterial activity compared with rGO and rGO-S. Our data also showed that at concentrations below 150 μg/mL, rGO-S/Se displayed negligible inhibitory action on *S. aureus*. (Figure 3C). It is speculated that rGO alone below 100 μg/mL will have antibacterial activity ≤ 23% (Figure 3A). S, Se, and S/Se NPs at similar concentrations of 100 μg/mL, 150 μg/mL, and 200 μg/mL did not show any antibacterial effect against *S. aureus* (Figure S2).

**Figure 4:**
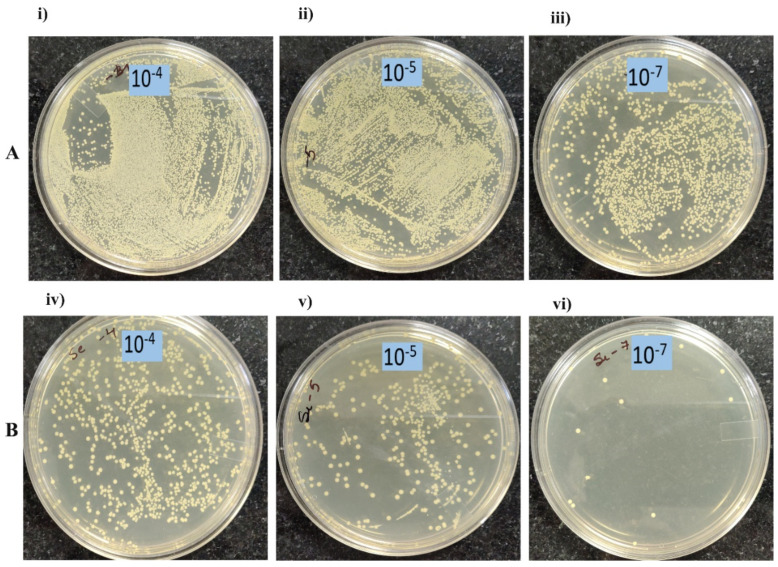
The viability of *S. aureus* bacterial cells with rGO-S/Se 200 μg/mL NPs after 8 h incubation. Photographs of *S. aureus* (**A**) without NPs and (**B**) with NPs of rGO-S/Se, at (i), (iv) 10^−4^; (ii), (v) 10^−5^; and (iii), (vi) 10^−7^ dilutions.

To further confirm that cell viability is lost upon treatment with nanoparticle rGO-S/Se, we performed colony-forming unit (CFU) assay. As evident in Figure 4B, *S. aureus* exposed to 200 μg/mL rGO-S/Se showed a sharp decline in the number of viable cells when compared with untreated *S. aureus* samples at higher dilutions (Figure 4A). Taken together, our results suggested that rGO-S/Se NP have the most antibacterial activity against *S. aureus*.

**Figure 5:**
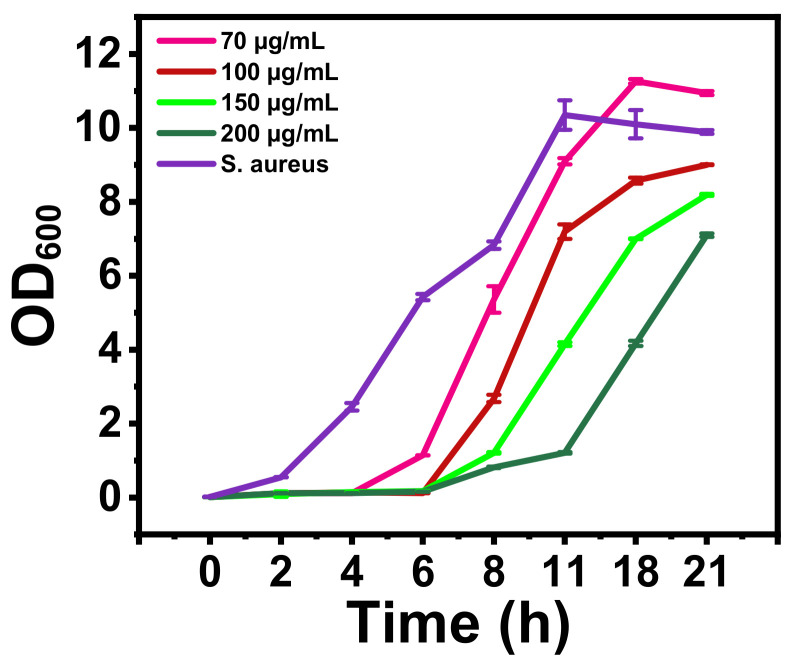
The effect of rGO-S/Se NPs on the growth of *S. aureus* in TSB containing various concentrations of NPs (70 μg/mL, 100 μg/mL, 150 μg/mL, 200 μg/mL, and *S. aureus* without NPs as control) grown at 37 °C with shaking. The growth curve was determined by measuring the OD_600_ nm of the sample versus time (h). Error bar represents standard error of mean.

Previous studies indicated that Se NPs have more bacteriostatic effect than bactericidal action on microbes [31,56,57]. To prove whether rGO-S/Se also have a bacteriostatic mode of action, growth studies on *S. aureus* treatment with varying concentrations of rGO-S/Se NPs were performed (Figure 5). rGo-S/Se at 200 μg/mL completely impeded the growth of *S. aureus* until 6 h only. After 6 h, *S. aureus* cells were able to resume growth as observed by an increase in optical density. Surprisingly, *S. aureus* cells were able to resume growth at high rGO-S/Se NPs concentrations comparable to untreated control cells (Figure 5), indicating that rGO-S/Se NPs may have a bacteriostatic effect. However, these results should be interpreted with caution as with increased time NPs tend to aggregate, which leads to a decrease in effective concentrations well below MIC and may be the reason for the bacteriostatic action observed [58].

**Figure 6:**
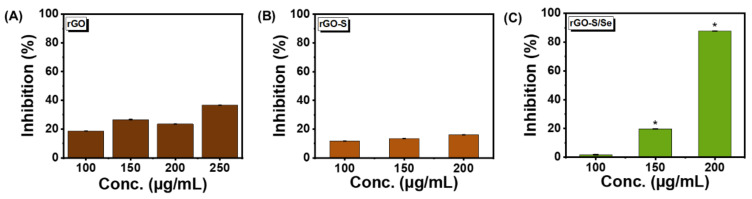
Effect of rGO, rGO-S, and rGO-S/Se on *E. faecalis* growth. Percentage of bacterial growth inhibition when exposed to varying concentrations of (**A**) rGO, (**B**) rGO-S, and (**C**) rGO-S/Se; * *p*-value < 0.01. Error bar represents the standard error of the mean.

We further tested the antibacterial activity of rGO-S/Se against other Gram-positive pathogens, including *Enterococcus faecalis* (*E. faecalis*), which has emerged as one of the leading cause of nosocomial infections [59]. Antibacterial activity of rGO, rGO-S, and rGO-S/Se NPs were tested against *E. faecalis.* The bacterial growth was inhibited only 20% at 150 μg/mL but was drastically increased (87%) when 200 μg/mL concentration of rGO-S/Se was used (Figure 6C). In comparison, rGO and rGO-S NPs did not exhibit efficient antibacterial activity against. *E. faecalis* even at high concentrations of 200 μg/mL (Figure 6A,B), suggesting that in the case of *E. faecalis*, rGO-S/Se NPs have more antibacterial potential.

Bactericidal action of NPs is dependent on particle size, concentrations, and exposure time. Many researchers have obtained similar results pertaining to nanoparticle antimicrobial activity [60]. Sengupta et al. observed maximum growth inhibition of 48.6% in *S. aureus* with rGO at the concentration of 3 mg/mL [61]. The smaller size and spherical shape of *S. aureus* results in its lesser surface area of exposure to rGO nanomaterial becoming the main cause of low bacterial growth inhibition on exposure to rGO, signifying the influence of bacterial shape on nanomaterial activity [62]. Since rGO NPs alone were not effective against *S. aureus*, a combination of NPs would enhance the antimicrobial efficacy of the material. Though S NPs have been reported as antimicrobial agents, those reports are very few and inconsistent [30,35]. Selenium NPs displayed significantly less antimicrobial activity against Gram-positive bacteria *S. aureus* and *B. subtilis* compared with Gram-negative *E.coli* and *P. aeruginosa* [63]. Rangrazi et al. reported similar observation of MICs of 137 and 274 μg/mL against *S. aureus* and *E. faecalis,* respectively, while testing the antimicrobial activity of chitosan-stabilized Se NPs [64]. As rGO-S/Se NPs showed consistent and robust antibacterial activity, further studies were performed on these NPs only.

### 3.4. rGO-S/Se NPs Generate Oxidative Stress

**Figure 7:**
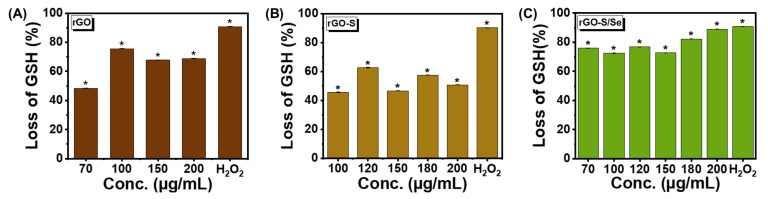
Concentration dependent oxidative stress induced by NPs. Loss of glutathione percentage after in vitro incubation with the (**A**) rGO; * *p*-value < 0.05 (**B**) rGO-S; * *p*-value < 0.005 and (**C**) rGO-S/Se; * *p*-value < 0.01, at different concentrations ranging from 70 μg/mL to 200 μg/mL for 3 h. H_2_O_2_ was used as a positive control. Error bar represents standard error of mean.

One of the major reasons for the toxicity of NPs towards bacterial cells is attributed to an increase in oxidative stress. NPs are believed to create oxidative stress in bacteria by causing an increase in reactive oxygen species (ROS) such as hydrogen peroxide (H_2_O_2_), reactive superoxide anion radicals (O^2−^), and hydroxyl radicals (ȮH), which leads to ROS mediated oxidative stress in the bacterial cell. ROS causes DNA base oxidation, protein carbonylation and lipid peroxidation [47,65,66,67]. In our study rGO-S/Se NPs exhibited robust antibacterial activity against Gram-positive pathogens, and we investigated further whether this toxicity was due to oxidative stress. For determination of oxidative stress potential of NPs, abiotic glutathione (GSH) oxidation assay was performed. GSH, a tripeptide with thiol groups, serves as a non-enzymatic antioxidant system. Thiol groups present in glutathione are oxidized to form glutathione disulfide (GSSG), a redox reaction that protects cells from oxidative damage by scavenging the ROS generated [20,36,68]. The amount of free thiol groups of GSH present was determined by Ellman’s reagent, which forms a yellow-colored product due to 2-nitro-5-thiobenzoic acid (NTB) upon reaction with the free thiol group. All three NPs rGO, rGO-S, and rGO-S/Se were tested. Figure 7 shows the loss of GSH when exposed to rGO, rGO-S, and rGO-S/Se NPs with concentrations ranging from 70 μg/mL to 200 μg/mL. Increasing rGO concentrations from 70 μg/mL to 200 μg/mL led to increased GSH loss with a maximum value of up to 76% (Figure 7A). H_2_O_2_ was used as a positive control which resulted in 91% GSH loss. At the concentration of 100 μg/mL, rGO-S resulted in only a 45% decrease in GSH. Maximum GSH loss (62%) was observed with 120 μg/mL of rGO-S. However, further increase in the concentration of nanomaterial resulted in decreased levels of GSH oxidation (Figure 7B). The decrease in GSH loss at high rGO-S NP concentrations could be due to the agglomeration of NPs [69]. As evident from Figure 7C, rGO-S/Se NPs showed maximum GSH oxidation (72%) at concentration ≥70 μg/mL indicating that rGO-S/Se is able to efficiently generate oxidative stress. In comparison, exposure at lower concentrations of both rGO and rGO-S did not generate a robust oxidative stress response. rGO-S NPs generated the least GSH loss, which is in accordance with its diminished toxicity towards bacterial cells.

As determined from TGA, the loading of NPs was ~80% on the rGO surface, signifying rGO was present at 20% only in both the synthesized composites. The loss of GSH by rGO alone was 47% at 70 μg/mL and Figure 3A indicates inhibition of bacteria growth at 100 μg was only 25%. This suggests rGO-related inhibition activity was minimal in the case of rGO/S-Se nanocomposites. The observed GSH values were mainly due to S/S-Se NPs only.

**Figure 8:**
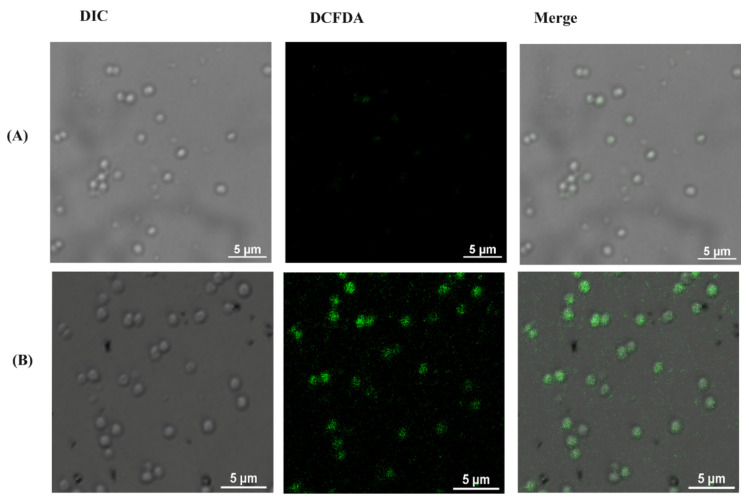

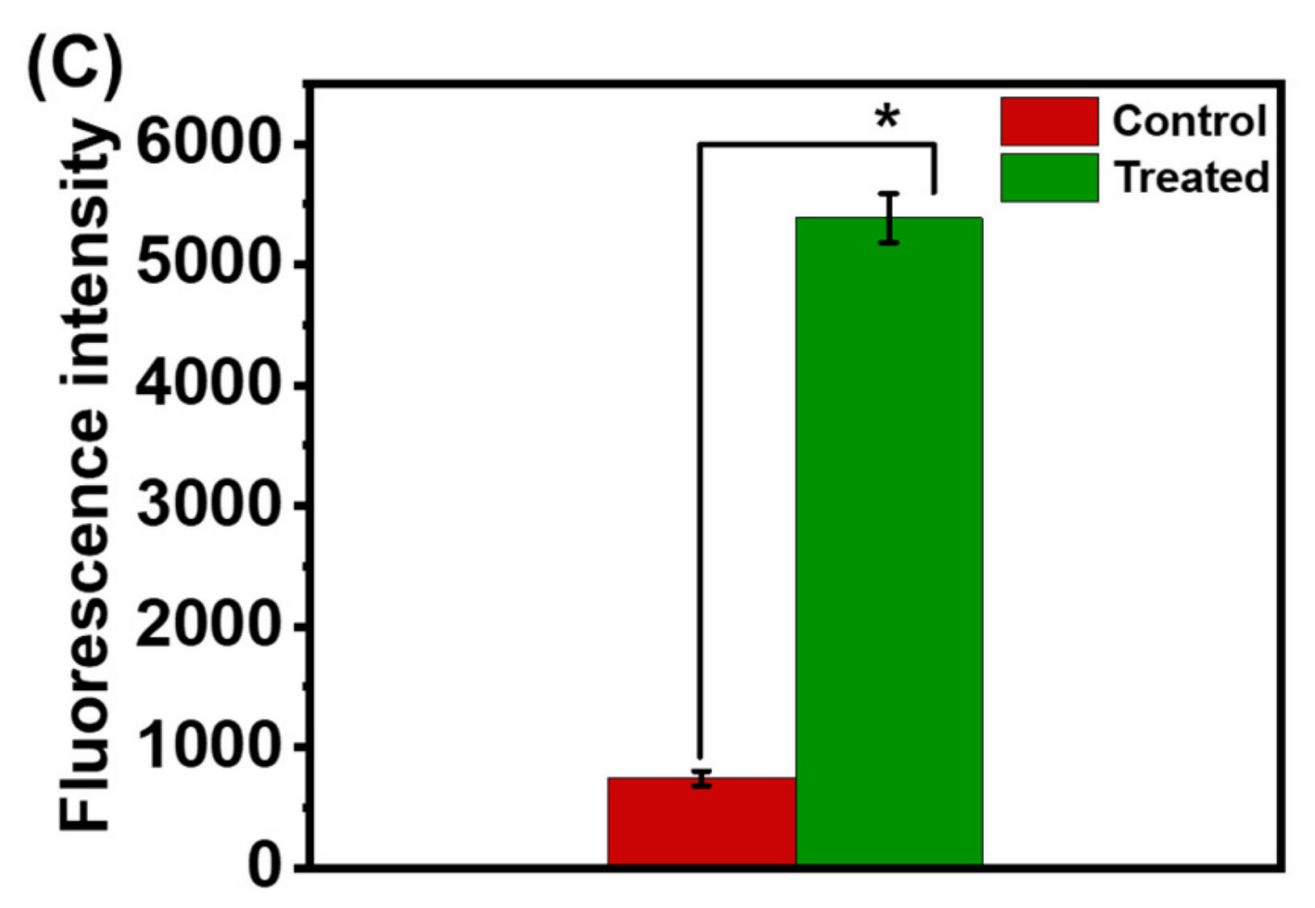
Intracellular ROS was measured by DCFDA staining after rGO-S/Se (200 μg/mL) treatment. Representative fluorescence images of *S. aureus* (**A**) without NPs and (**B**) with NPs. (**C**) is the quantified data of (**A**,**B**) where the control is *S. aureus* without NPs and treated is *S. aureus* after 8 h of NPs exposure, Scale bar = 5 μm. The intensity of the green fluorescence indicates ROS concentration in the cells. Error bar represents the standard error of the mean. * *p*-value < 0.001.

Since we observed maximum bacterial growth inhibition and high GSH oxidation with rGO-S/Se NPs, increase in biotic oxidative stress was also probed. ROS generated in cells can be quantified by using oxidant sensing DCFDA which on reaction with ROS was later oxidized into fluorescent 2′, 7′-dichlorofluorescein (DCF). Untreated cells (Figure 8A) displayed negligible fluorescence, while a significant increase in DCF fluorescence intensity was observed in rGO-S/Se (200 μg/mL)-treated *S. aureus* bacterial cells (Figure 8B), suggesting ROS generation in these cells. Figure 8C shows the quantified DCFDA fluorescence intensity, which clearly depicts increased DCFDA intensity in treated cells. A similar increase in ROS was observed in *E. faecalis* treated with rGO-S/Se at the same concentration (Figure S3). Based on the above findings, it can be concluded that increased oxidative stress is one of the reasons contributing towards bacterial toxicity for rGO-S/Se NPs.

Our results are consistent with previous findings as similar mechanisms of action of rGO NPs have been reported [70,71]. Zheng et al. reported increased ROS generation in Gram-positive microorganisms after treatment with selenium NPs. [72]. ROS generation by rGO NPs lead to cell death in *Pseudomonas aeruginosa* [73,74]. Furthermore, ROS generation by Se NPs in *S. aureus* is heavily dependent on particle size [75]. *S. aureus* cells are spherical and have reduced surface area compared with rod-shaped cells, which affect NPs interaction with the bacterium [60].

### 3.5. Exposure to rGO-S/Se NPs Leads to Morphological Alterations

**Figure 9:**
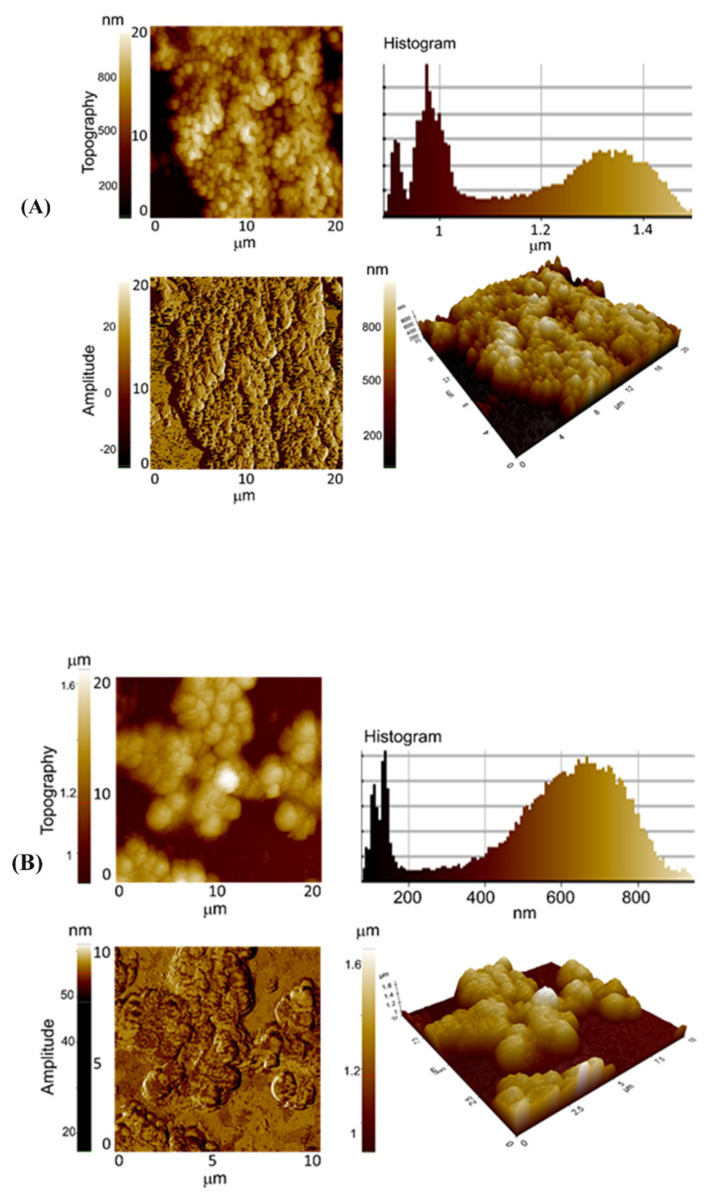
Representative AFM (topography, histogram, 3D, and amplitude) images of *S. aureus* after incubation with the 200 μg/mL rGO-S/Se NPs. *S. aureus* in the absence of NPs was taken as the positive control. (**A**,**B**) *S. aureus* after 8 h incubation with rGO-S/Se NPs, Scale bar = 10 μm.

NPs are known to cause cell wall and membrane damage in bacterial cells. Adsorption and penetration of NPs lead to membrane depolarization, permeability changes, and cell lysis [20]. To analyze membrane perturbations, AFM analysis of *S. aureus* was performed before and after treatment with rGO-S/Se NPs. Figure 9 shows the effects on the morphology of *S. aureus* after exposure to rGO-S/Se NPs. Untreated bacterial cells displayed regular spherical shapes and smooth surfaces (Figure 9A), whereas NPs-exposed cells formed aggregates (Figure 9B). After exposure of *S. aureus* to NPs, significant morphological aberrations were observed. NP-treated cells displayed less distinct edges with loss of coccoid shape, and formed aggregates (Figure 9B). Our results are in congruence with previous reports, as bare selenium NPs and sharp edges of rGO are reported to cause cell membrane disruption and deformation [57,62,76].

### 3.6. Adsorption of rGO-S/Se on the Bacterial Cell Surface

**Figure 10:**
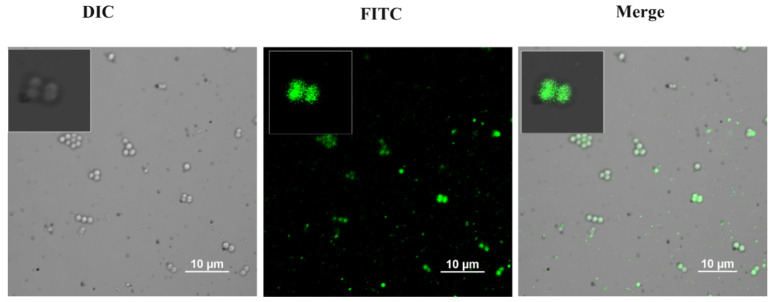
CLSM images of *S. aureus* cells after 8 h incubation with FITC-rGO-S/Se NPs (100 μg/mL). Scale bar = 10 μm.

Nanomaterial-bacterial interactions vary depending on the type of graphene nanomaterial/compositions used. rGO nanomaterials are hydrophobicand tend to strongly adsorb on bacterial surfaces. Physical contact between NPs and bacterial cells is proposed to be crucial for antibacterial activity and could be achieved by electrostatic attraction, van der Waals forces, and hydrophobic interactions [77,78,79]. The interaction or uptake of NPs greatly influences their antimicrobial efficiency, which could be detected and quantified using various techniques such as electron microscopy and fluorescence-based techniques involving confocal microscopy (CLSM) and flow cytometry. Among these, CLSM using fluorescence-labelled NPs is more commonly used [80,81,82]. We generated a fluorophore FITC-tagged variant of rGO-S/Se NP to study its interaction with *S. aureus* cells. FITC-tagged rGO-S/Se NPs were exposed to bacterial cells and analyzed by CLSM. *S. aureus* incubated with FITC-tagged rGO-S/Se (100 μg/mL) showed high fluorescence intensity throughout the cell (Figure 10). Most NPs were associated with bacterial cells and were not detached even after washing. It should be noted that at a concentration above 100 μg/mL FITC-tagged rGO-S/Se NPs caused severe damage to bacterial cells (data not shown). Our findings indicated that rGO-S/Se NPs get deposited onto cell surfaces and directly interact with the bacterial cells.

To date, significant reports suggest the contact between nanomaterials and bacterial cells as a crucial factor governing antibacterial function [19]. The chemical and physical properties of the nanomaterial dictate their selective antimicrobial actions towards Gram-positive or Gram-negative microbes. For example, zinc oxide and nickel oxide NPs inhibit *S. aureus* and *B. subtilis* whereas AgNPs exhibit antimicrobial activity against Gram-negative *E. coli* and *Pseudomonas aeruginosa* [1]. Similarly, *E. coli* are susceptible to copper oxide NPs but has less effects on the growth of *S. aureus* and *B. subtilis* [83,84]. However, toxicity of AgNPs and CuNPs is heavily influenced by growth media, and their toxicity in DI water is 100–10,000 times higher than in rich growth media [84]. Gram-positive and Gram-negative bacteria differ significantly in their cell wall structure, which influences bacterial-nanomaterials interactions. Gram-negative bacteria have a complex wall structure with a thin layer of peptidoglycan sandwiched between two phospholipid membranes. The outer membrane is predominantly composed of lipopolysaccharides carrying negative charge, thus enhancing its affinity towards positive ions released by NPs. In contrast, Gram-positive bacteria have a thick peptidoglycan layer with covalently attached teichoic acids which prevents aggregation of NPs and keeps them spread out along phosphate groups [17,83]. Caudil et al. demonstrated the influence of teichoic acids and their component in gold NPs adhesion, where the extent of interaction observed was more in wall teichoic acids from the wild type when compared with teichoic acids lacking alanine [85]. However, both Gram-positive and Gram-negative bacteria have a negatively charged cell wall, enhancing the attraction between NPs and bacterial cells [20]. Understanding interactive relationships between nanomaterials and bacteria would help the in modulation of nanomaterials and inhibit bacterial growth without any adverse effects.

## 4. Conclusions

In this study, the antibacterial potentials of three synthesized rGO, rGO-S, and rGO-S/Se nanoparticles were compared against Gram-positive pathogens *S. aureus* and *E. faecalis.* rGO-S/Se NPs demonstrated exceedingly robust antibacterial activity both in suspension MIC and CFU assays. Growth curve analysis revealed concentration and time-dependent bacteriostatic activity of the rGO-S/Se NP against *S. aureus* cells. Further investigation revealed that antibacterial action is attributed to ROS generation by rGO-S/Se NPs. rGO-S/Se NPs probably have multiple modes of action, which include process physical contact or adsorption on the bacterial cell and membrane perturbations, followed by oxidative stress generation in the cell. Our studies elucidated the antimicrobial potential of rGO and rGO-S/Se nanocomposites and further inquiry in this direction would be beneficial in the development of biomedical applications.

## Data Availability

All data can be found in this paper or in papers cited here.

## Supplementary Materials

The following supporting information can be downloaded at: https://www.mdpi.com/article/10.3390/nano12020191/s1, Figure S1: Powder X-ray diffraction of sulfur, selenium and S/Se alloy itle; Figure S2: Effect of S, Se and S/Se NPs on S. aureus growth; Figure S3: Intracellular ROS was measured by DCFDA staining after rGO-S/Se (200 µg/ mL) treatment.
