# Supplementary material for: Synthesis and Characterization of Sulfur and Sulfur-Selenium Nanoparticles Loaded on Reduced Graphene Oxide and Their Antibacterial Activity against Gram-Positive Pathogens
Source: Nanomaterials (Basel). 2022 Jan 7;12(2):191. doi: 10.3390/nano12020191 (PMC8782023; doi:10.3390/nano12020191)
Supplement: Supplementary file 1 [file nanomaterials-12-00191-s001.zip › nanomaterials-1497658-supplementary.pdf]

nanomaterials

Supplementary Materials

Synthesis and Characterization of Sulfur and Sulfur-Selenium Nanoparticles Loaded on Reduced Graphene Oxide and Their Antibacterial Activity against Gram-positive Pathogens

Rashmi Niranjan [1,†], Saad Zafar [2,†], Bimlesh Lochab [2,*] and Richa Priyadarshini [1,*]

1 Department of Life Sciences, School of Natural Sciences, Shiv Nadar University, Gautam Buddha Nagar 201314, India; rn161@snu.edu.in
2 Materials Chemistry Laboratory, Department of Chemistry, School of Natural Sciences, Shiv Nadar University, Gautam Buddha Nagar 201314, India; sz539@snu.edu.in
* Correspondence: bimlesh.lochab@snu.edu.in (B.L.); richa.priyadarshini@snu.edu.in (R.P.)
† These authors contributed equally to this work.

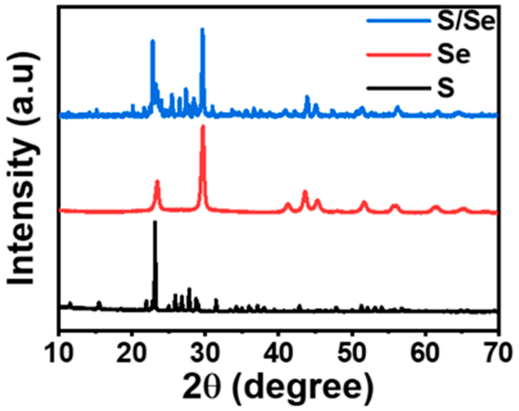

Figure S1. Powder X-ray diffraction of sulfur, selenium and S/Se alloy.

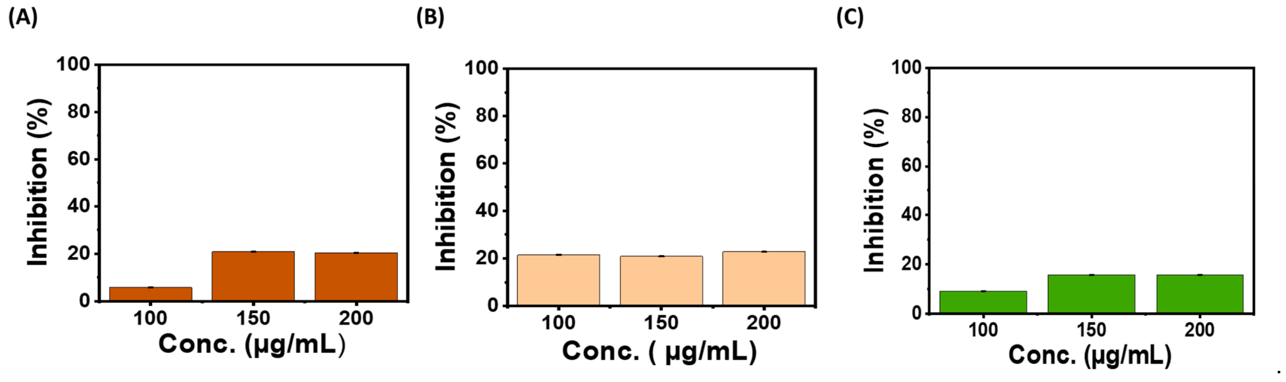

Figure S2. Effect of S, Se and S/Se NPs on *S. aureus* growth. Percentage of bacterial growth inhibition when exposed to varying concentrations of (A) S; (B) Se; and (C) S/Se. Error bar represents standard error of mean.

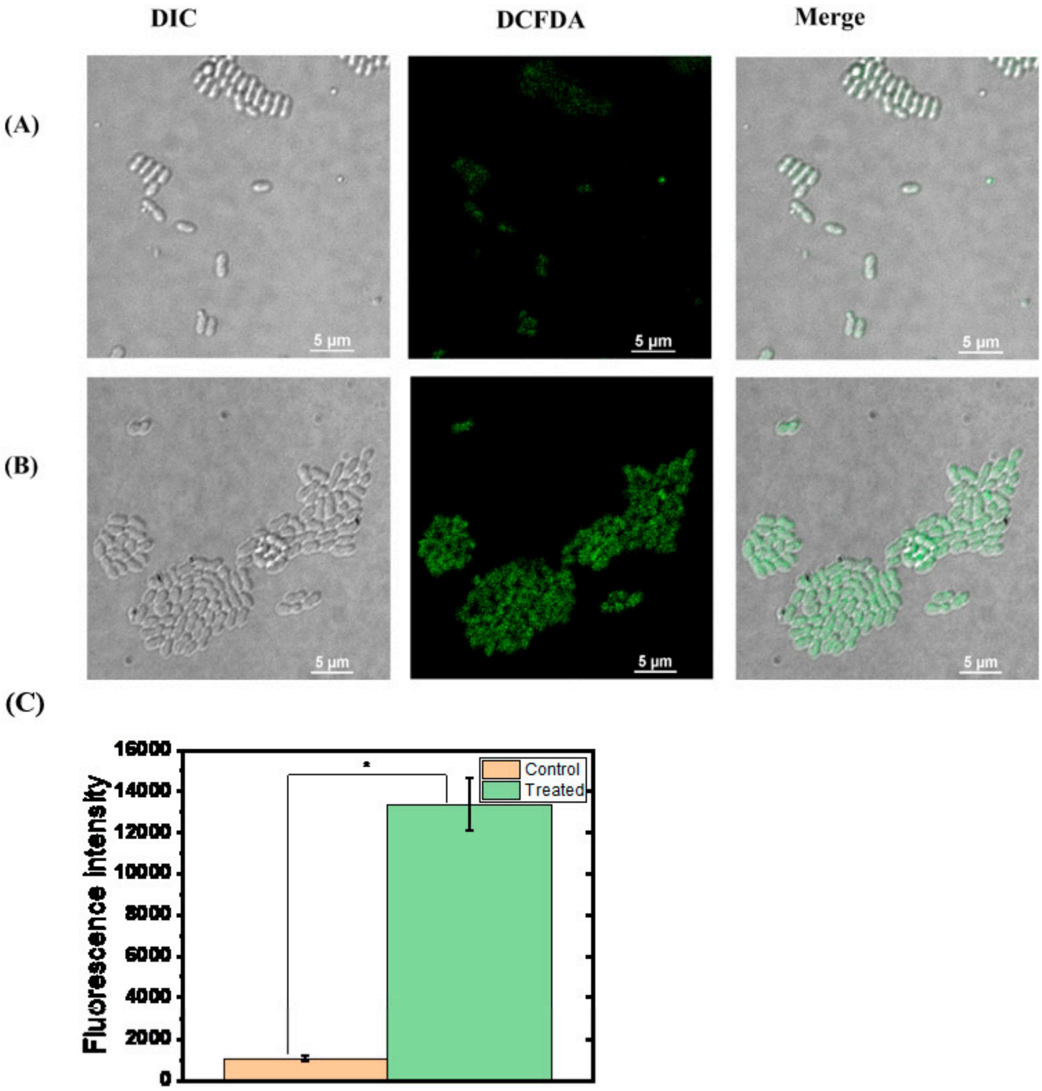

Figure S3. Intracellular ROS was measured by DCFDA staining after rGO-S/Se (200 µg/ mL) treatment. Representative fluorescence images of *E. faecalis* (A)without NPs and (B)with NPs (C) is the quantified data of (A) and (B) where control is *E. faecalis* without NPs and treated is *E. faecalis* after 8 h of NPs exposure, Scale bar = 5 µm. The intensity of the green fluorescence indicates ROS concentration in the cells. Error bar represents the standard error of the mean. *p-value < 0.001.
